# Plasma Aβ and neurofilament light chain are associated with cognitive and physical function decline in non-dementia older adults

**DOI:** 10.1186/s13195-020-00697-0

**Published:** 2020-10-08

**Authors:** Lingxiao He, Philipe de Souto Barreto, Geetika Aggarwal, Andrew D. Nguyen, John E. Morley, Yan Li, Randall J. Bateman, Bruno Vellas, For the MAPT/DSA Group, Bruno Vellas, Sophie Guyonnet, Isabelle Carrié, Lauréane Brigitte, Catherine Faisant, Françoise Lala, Julien Delrieu, Hélène Villars, Emeline Combrouze, Carole Badufle, Audrey Zueras, Sandrine Andrieu, Christelle Cantet, Christophe Morin, Gabor Abellan Van Kan, Charlotte Dupuy, Yves Rolland, Céline Caillaud, Pierre-Jean Ousset, Françoise Lala, Sherry Willis, Sylvie Belleville, Brigitte Gilbert, Jean-François Dartigues, Isabelle Marcet, Fleur Delva, Alexandra Foubert, Sandrine Cerda, Corinne Costes, Olivier Rouaud, Patrick Manckoundia, Valérie Quipourt, Sophie Marilier, Evelyne Franon, Lawrence Bories, Marie-Laure Pader, Marie-France Basset, Bruno Lapoujade, Valérie Faure, Michael Li Yung Tong, Christine Malick-Loiseau, Evelyne Cazaban-Campistron, Françoise Desclaux, Colette Blatge, Thierry Dantoine, Cécile Laubarie-Mouret, Isabelle Saulnier, Jean-Pierre Clément, Marie-Agnès Picat, Laurence Bernard-Bourzeix, Stéphanie Willebois, Iléana Désormais, Noëlle Cardinaud, Marc Bonnefoy, Pierre Livet, Pascale Rebaudet, Claire Gédéon, Catherine Burdet, Flavien Terracol, Alain Pesce, Stéphanie Roth, Sylvie Chaillou, Sandrine Louchart, Kristelle Sudres, Nicolas Lebrun, Nadège Barro-Belaygues, Jacques Touchon, Karim Bennys, Audrey Gabelle, Aurélia Romano, Lynda Touati, Cécilia Marelli, Cécile Pays, Philippe Robert, Franck Le Duff, Claire Gervais, Sébastien Gonfrier, Yannick Gasnier, Serge Bordes, Danièle Begorre, Christian Carpuat, Khaled Khales, Jean-François Lefebvre, Samira Misbah El Idrissi, Pierre Skolil, Jean-Pierre Salles, Carole Dufouil, Stéphane Lehéricy, Marie Chupin, Jean-François Mangin, Ali Bouhayia, Michèle Allard, Frédéric Ricolfi, Dominique Dubois, Marie Paule Bonceour Martel, François Cotton, Alain Bonafé, Stéphane Chanalet, Françoise Hugon, Fabrice Bonneville, Christophe Cognard, François Chollet, Pierre Payoux, Thierry Voisin, Julien Delrieu, Sophie Peiffer, Anne Hitzel, Michèle Allard, Michel Zanca, Jacques Monteil, Jacques Darcourt, Laurent Molinier, Hélène Derumeaux, Nadège Costa, Bertrand Perret, Claire Vinel, Sylvie Caspar-Bauguil, Pascale Olivier-Abbal, Sandrine Andrieu, Christelle Cantet, Nicola Coley

**Affiliations:** 1 Gérontopôle de Toulouse, Institut du Vieillissement, Centre Hospitalo-Universitaire de Toulouse, 37 allées Jules Guesdes, 31000 Toulouse, France; 2 UMR UPS/INSERM, 1027 University of Toulouse III, Toulouse, France; 3 Division of Geriatric Medicine, Saint Louis University School of Medicine, St. Louis, MO USA; 4 Henry and Amelia Nasrallah Center for Neuroscience, Saint Louis University, St. Louis, MO USA; 5 Department of Neurology, Washington University School of Medicine, 660 South Euclid Avenue, Box 8111, St. Louis, MO 63110 USA

**Keywords:** Amyloid-β, Neurofilament light chain, Cognitive function, Physical function

## Abstract

**Background:**

Cognition is closely associated with physical function. Although high brain amyloid-β (Aβ) deposition and neurofilament light chain (NfL) are associated with cognitive and gait speed decline, relationships of combined plasma Aβ and NfL profiles with cognitive and physical functions in older adults remain unknown. The research aim of this study was to investigate the prospective associations of combined plasma Aβ and NfL profiles with cognitive and physical functions in older adults.

**Methods:**

Participants (*n* = 452, aged 76 ± 5 years) who had both plasma Aβ and NfL data collected from the Multidomain Alzheimer’s Preventive Trial (MAPT, May 2008 to April 2016) were included in the current study. These participants were from four MAPT groups (multidomain interventions [physical activity and nutritional counselling, and cognitive training], omega-3 supplementation, multidomain plus omega-3 supplementation and control group) and had received a 3-year intervention, followed by a 2-year observational follow-up. Cognitive function was evaluated as Mini-Mental State Examination and composite cognitive score (CCS, a mean *Z*-score combining four cognitive tests). Physical function was evaluated as gait speed (4-m usual-pace walk test) and chair-stand time (5-time maximal chair-stand test). Cognitive and physical function data measured at the time of and after blood Aβ and NfL tests were used for analysis. Participants with plasma Aβ_42_/Aβ_40_ ratios lower than 0.107 and NfL levels greater than 93.04 pg/ml were classified as Aβ+ and NfL+. Multivariable regressions and mixed-effects linear models were used for the analysis.

**Results:**

At the cross-sectional level, no significant association was found between Aβ+NfL+ and cognitive or physical function after controlling for age, sex, body mass index, education level and MAPT group. Evaluating longitudinal changes, participants with Aβ+NfL+ had greater annual declines in the CCS (*β* = − 0.11, 95%CI [− 0.17, − 0.05]) and gait speed (*β* = − 0.03, 95%CI [− 0.05, − 0.005]). After adjusting for APOE ɛ4 genotype, Aβ+NfL+ was associated with a greater decline only in the CCS (*β* = − 0.09, 95%CI [− 0.15, − 0.02]).

**Conclusions:**

Combined low plasma Aβ_42_/Aβ_40_ ratio and high plasma NfL level was associated with greater declines in cognition and gait speed over time, providing further evidence of the links between cognitive and physical function.

**Trial registration:**

www.clinicaltrials.gov [NCT00672685].

## Background

Decreased physical function has been associated with cognitive impairment in older adults [1, 2]. Multiple studies have examined the association between physical function and brain biomarkers of neurodegeneration, such as amyloid-β (Aβ) and neurofilament light chain (NfL). Higher brain Aβ burden, cerebrospinal fluid (CSF) Aβ_42_/Aβ_40_ ratio and NfL levels are associated with slower gait speed [3–5] and greater gait variability [6]. Yet, no association was found with chair-stand [7]. Considering the close connection between brain and plasma biomarkers, it is plausible to think that plasma Aβ and NfL concentrations might also be associated with physical function. However, no study has been performed to examine this association in an older population. Meanwhile, a recent research by de Wolf et al. [8] has reported that adults with both low plasma Aβ_42_ and high NfL levels demonstrated a higher risk of developing dementia or AD than those with one biomarker condition (i.e. low Aβ_42_ or high NfL). Therefore, a combined plasma Aβ and NfL condition might be associated with declines in both cognitive and physical functions. The objective of this study is to explore the possible association between combined plasma Aβ and NfL condition with the evolution of physical function in older adults.

## Methods

The Multidomain Alzheimer’s Preventive Trial (MAPT, ClinicalTrials.gov [NCT00672685]) was a randomized, controlled trial that compared multidomain interventions (physical activity and nutritional counselling and cognitive training) and omega-3 supplementation, combined or alone, with a placebo control group [9]; no effects of the MAPT interventions on cognitive function and gait speed over a 3-year period were found [10]. MAPT was approved by the ethics committee in Toulouse (CPP SOOM II). Written consent forms were obtained from all participants.

### Study population

Older adults (70 years or over) with spontaneous memory complaints or limitations in at least one instrumental activity of daily living or a gait speed lower than 0.8 m/s were recruited in the MAPT. Our current study included 452 participants who had available information on plasma Aβ and NfL levels.

### Evaluation of cognitive and physical functions

Cognitive function was evaluated as Mini-Mental State Examination (MMSE, ranging from 0 to 30, higher is better) and composite cognitive score (CCS), which was calculated as a mean *Z*-score combining four cognitive tests (free and total recall of the Free and Cued Selective Reminding Test, ten MMSE orientation items, the Digit Symbol Substitution Test score from the Wechsler Adult Intelligence Scale–Revised and the Category Naming Test) [10]. Physical function was evaluated as gait speed, which was measured by a 4-m usual-pace walk test (m/s), and chair-stand time (s), which was measured by a 5-repetition maximal speed chair-stand test. Outcome measures were evaluated at baseline, the 6th month and each year during the 3-year intervention period and the 2-year observational follow-up. Data of outcome measures collected at the same time point and after blood tests were used in this study.

### Measurement of plasma Aβ and NfL levels

Plasma Aβ_42_ and Aβ_40_ levels were assessed by immunoprecipitation mass spectrometry as previously described [11]. Aβ levels were analysed and calculated by integrated peak area ratios to known concentrations of the internal standards using the Skyline software package [12]. Plasma NfL levels were measured using the R-PLEX human neurofilament L antibody set (Meso Scale Discovery, F217X). Samples were diluted 2-fold in a diluent buffer and tested in duplicate according to the manufacturer’s instructions.

Plasma Aβ or NfL levels were measured once using blood samples collected in the first or second year of the study. For most participants (*n* = 420 out of 452), Aβ and NfL levels were measured from blood samples taken at the 1-year wave of data collection. For the rest of the participants, plasma markers were measured using samples from the second year. Blood samples were collected on the same day as cognitive and physical function evaluation.

### Plasma Aβ_42_/Aβ_40_ ratio and NfL stratification

The selection of Aβ_42_/Aβ_40_ ratio cut-off value was performed based on participants (*n* = 201) who underwent amyloid [^18^F] florbetapir positron emission tomography (PET positive when standardized uptake value ratios ≥ 1.17) [13] using logistic regression (PET positive/negative as the dependent variable and Aβ_42_/Aβ_40_ as the independent variable) with Youden’s Index as the metric (higher is better) [11]. The cut-off value of NfL was determined as the upper quartile of our participants. Participants with low Aβ_42_/Aβ_40_ ratios (≤ 0.107) and high NfL levels (≥ 93.04 pg/ml) were classified as Aβ+NfL+.

### Covariates

Age, sex, body mass index (kg/m^2^), education level and MAPT group. Since almost 10% of the participants did not have APOE ɛ4 genotype data, separate models were performed with and without APOE ɛ4 genotype (dichotomous variable) as an additional covariate.

### Statistics

Descriptive data was presented as mean ± standard deviation (SD) or absolute numbers with percentage, as appropriate. Comparisons between the Aβ+NfL+ group and the rest of the population were performed with Student’s *t* test and chi-square test, as appropriate.

Multivariable linear regression was used for cross-sectional analyses with cognitive or physical functions (separate models) measured at the moment of the blood test as dependent variables and Aβ+NfL+ as the independent variable of interest. Longitudinal analyses were performed using separated mixed-effects linear models with longitudinal data of cognitive or physical functions as dependent variables. A random effect at the participant level and a random slope of time were assumed. All analyses were controlled for the covariates mentioned above and were performed using SAS 9.4. *p* values were corrected for multiple testing by Benjamini–Hochberg procedure [14], with a significance level of 0.05, in cross-sectional and longitudinal models with and without APOE ɛ4 genotype, separately.

## Results

The characteristics of the participants are presented in Table 1. The average age of our participants was 76 years, and 59% of the participants were female. ninety-three per cent of the participants had plasma biomarkers tested from blood samples collected 1 year after the enrolment of the MAPT. Participants with plasma Aβ+NfL+ were older and had lower CCS than the other participants.

**Table 1:** Characteristics of participants in this study

Parameters	All participants			Participants with APOE genotype data		
	Total (*n* = 452)	Aβ+NfL+ group (*n* = 46)	Other participants^†^ (*n* = 406)	Total (*n* = 410)	Aβ+NfL+ group (*n* = 39)	Other participants^†^ (*n* = 371)
Age (year), mean (SD)	76 (5)	78 (5)**	76 (5)	76 (5)	78 (5)**	76 (5)
Female, *n* (%)	268 (59%)	23 (50%)	245 (60%)	224 (55%)	19 (49%)	224 (60%)
Participants with plasma biomarkers measured in the first year, *n* (%)	420 (93%)	44 (96%)	376 (93%)	382 (93%)	37 (95%)	345 (93%)
Initial body mass index, mean (SD)	26.4 (4.0)	25.9 (3.8)	26.5 (3.9)	26.4 (3.8)	25.6 (3.1)	26.5 (3.9)
Initial CCS, mean (SD)	0.07 (0.79)	− 0.25 (0.70)**	0.10 (0.79)	0.10 (0.77)	− 0.20 (0.71)*	0.14 (0.78)
Initial MMSE, mean (SD)	27.8 (1.9)	27.5 (1.7)	27.9 (1.9)	27.9 (1.8)	27.7 (1.6)	27.9 (1.8)
Initial gait speed (m/s), mean (SD)	1.0 (0.2)	1.0 (0.3)	1.0 (0.2)	1.0 (0.2)	1.0 (0.3)	1.0 (0.2)
Initial chair-stand time (s), mean (SD)	12.1 (4.3)	12.9 (4.0)	12.0 (4.4)	12.0 (4.3)	12.9 (4.2)	11.9 (4.3)
Aβ_42_/Aβ_40_ ratio, mean (SD)	0.114 (0.018)	0.097 (0.010)**	0.116 (0.018)	0.113 (0.018)	0.096 (0.010)**	0.115 (0.018)
NfL (pg/ml), mean (SD)	86.65 (74.09)	160.35 (137.01)**	78.30 (57.79)	85.70 (75.50)	162.55 (143.15)**	77.62 (59.23)

*p < 0.05, **p < 0.01 when compared Aβ+NfL+ group with other participants

^†^Other participants without Aβ+NfL+ condition

The cross-sectional analysis did not show any significant association between plasma Aβ+NfL+ and cognitive or physical function after controlling for covariates (Table 2).

**Table 2:** Multivariable linear regression of cognitive and physical functions by Aβ+NfL+ conditions

Parameters	Aβ+NfL+ group (all participants) ^#^				Aβ+NfL+ group (with APOE)^#^			
	Coefficient	*p*	Adjusted *p*^∆^	95%CI	Coefficient	*p*	Adjusted *p*^∆^	95%CI
Initial CCS	− 0.19	0.11	0.45	(− 0.42, 0.04)	− 0.15	0.24	0.85	(− 0.39, 0.10)
Initial MMSE	− 0.02	0.93	0.93	(− 0.60, 0.55)	− 0.03	0.92	0.92	(− 0.57,0.64)
Initial gait speed (m/s)	− 0.01	0.87	0.93	(− 0.08, 0.07)	− 0.02	0.63	0.85	(− 0.10, 0.06)
Initial chair-stand time (s)	0.34	0.61	0.93	(− 1.00, 1.69)	0.50	0.50	0.85	(− 0.95, 1.94)

Covariates: age, sex, body mass index, education level and MAPT group

^#^Participants without Aβ+NfL+ condition as the reference

^∆^*p* value adjusted by Benjamini–Hochberg procedure

Longitudinal analysis showed that participants with plasma Aβ+NfL+ had greater declines in CCS and gait speed over time. After adjusting for APOE ɛ4 genotype, Aβ+NfL+ was still associated with greater declines in CCS while no significance in gait speed was found (Table 3). No significant associations were found between Aβ+NfL+ status and MMSE or chair-stand. For the covariates, the MAPT intervention groups were not significantly associated with cognitive or physical functions.

**Table 3:** Mixed-effects linear models of cognitive and physical functions by Aβ+NfL+ conditions

Parameters	Aβ+NfL+ group × time (all participants)^#^				Aβ+NfL+ group × time (with APOE)^#^			
	Coefficient	*p*	Adjusted *p*^∆^	95%CI	Coefficient	*p*	Adjusted *p*^∆^	95%CI
CCS	− 0.11	0.0004	0.002	(− 0.17, − 0.05)	− 0.09	0.007	0.03	(− 0.15, − 0.02)
MMSE	− 0.14	0.18	0.24	(− 0.34, 0.06)	− 0.06	0.55	0.74	(− 0.27, 0.14)
Gait speed (m/s)	− 0.03	0.02	0.04	(− 0.05, − 0.005)	− 0.02	0.08	0.17	(− 0.05, 0.003)
Chair-stand time (s)	0.08	0.66	0.66	(− 0.28, 0.44)	− 0.001	0.99	0.99	(− 0.38, 0.38)

Covariates: age, sex, body mass index, education level and MAPT group

^#^Participants without Aβ+NfL+ condition as the reference

^∆^*p* value adjusted by Benjamini–Hochberg procedure

## Discussion

This study focused on the association between the combined plasma Aβ and NfL condition and cognitive and physical functions in older adults over a nearly 4-year period. We report for the first time that older adults with combined low plasma Aβ_42_/Aβ_40_ ratios and high NfL levels had more declines in cognitive (detected in CCS but not MMSE) and physical functions (detected in gait speed but not chair rise) over time while no cross-sectional association was found. Such findings indicate the feasibility of using plasma biomarkers to estimate the progressive decline of cognitive and physical functions in older adults over time.

Previous studies have reported the association between cognition and each of the biomarkers analysed in our research. Yaffe [15] analysed the baseline plasma Aβ_42_/Aβ_40_ ratio and a 10-year longitudinal cognition data in older adults (average age of 74 years) and reported that lower Aβ_42_/Aβ_40_ ratio was associated with greater cognitive decline. A similar longitudinal association between plasma NfL and cognition was also found in the study of Mielke et al. [16], who analysed the cognition data over a 15- or 30-month period in older adults with a median age of 76 years. Notably, no cross-sectional association between plasma biomarkers and cognition was found in both studies mentioned above. Our results based on combined plasma Aβ and NfL condition further confirmed these cross-sectional and longitudinal associations. Moreover, our findings are consistent with previous studies which reported that greater longitudinal cognitive declines were associated with higher plasma NfL levels in older adults with abnormal brain Aβ conditions (e.g. low CSF Aβ and high brain Aβ deposition) [17, 18].

Additionally, we further extend the literature by showing that the plasma Aβ+NfL+ profile was associated with the longitudinal decline of gait speed, but not chair-stand. These results are in line with our previous findings (based on the same MAPT dataset) about brain Aβ deposition and physical function, which reported that greater brain Aβ burden was associated with lower gait speed [3], but not chair rise [7]. Other researchers have also reported that high CSF Aβ_42_/Aβ_40_ ratio, NfL level and Aβ burden in the brain subregions were associated with increased gait variability [6] and low gait speed [4, 5], while similar studies for plasma Aβ and NfL are lacking. The difference in associations between gait speed and chair-stand with the plasma biomarkers in our study might be related to the various mechanisms behind those two physical functions. Chair-stand performance is more dependent on lower limb muscle function (strength and power) than gait speed; the latter is a more complex movement which involves dynamic balance control, coordination and muscle function. The significant association between plasma biomarkers and gait speed, but not chair-stand, indicates that these neurodegeneration-related biomarkers might be connected with the neural aspect of gait speed, such as cognitive status [19], motor control [20] and muscle synergies [21]. Since plasma NfL is closely associated with axonal damage [22] and plasma Aβ is associated with CSF Aβ [23], which is involved in synaptic function and multiple signalling pathways (e.g. calcium signalling and insulin-like growth factor-I signalling) [24], the less favourable plasma NfL and Aβ condition (i.e. Aβ+NfL+) might suggest structural and functional changes in the nervous system at a fundamental level. Consequently, besides cognitive degeneration, structural and functional changes in motor-related brain regions and peripheral neural pathways might also lead to gait speed decline. Therefore, further studies are still needed to explore the association between plasma biomarkers and the structural or functional changes in the central and peripheral nervous systems.

Notably, although Aβ+NfL+ was associated with gait speed decline, such decline has not reached the clinically meaningful threshold of 0.05 m/s [25]. Further studies are also needed to examine if the cognitive decline in CCS is clinically meaningful.

Our study only examined the initial plasma Aβ and NfL combined condition with cognitive and physical functions while previous studies have demonstrated the close associations between overtime biomarker changes and changes in cognitive performance. A 10-year follow-up study by Okereke et al. [26] on late middle-aged adults (average age of 64 years) reported that with a 1 SD (standard deviation) increase in the change of plasma Aβ_40_/Aβ_42_ ratio, there was a 0.02-unit/year more decline in global cognitive score. A recent study by Mattson et al. [27] has shown that increased plasma NfL levels over time were associated with larger global cognitive decline. Yet, the association between longitudinal biomarker changes and physical function remains to be established and needs further exploration.

## Limitation

We analysed data from a randomized controlled trial. Although there are no significant differences in plasma Aβ_42_/Aβ_40_ ratios and NfL levels among the MAPT groups, the MAPT interventions might affect plasma Aβ and NfL levels which were tested based on blood samples collected at the first/second year of the study. Therefore, it would be more appropriate if observational data were used. Since the longitudinal biomarker data were not available in our study, we could not confirm if there was any change in the combined Aβ and NfL status over the 4-year period. Moreover, the absence of longitudinal data of the biomarkers impeded us from looking at the association between changes in biomarkers and changes in cognitive and physical functions.

## Conclusions

Our results suggest the possibility of using easily accessible plasma Aβ_42_/Aβ_40_ ratio and NfL profiles as combined biomarkers to estimate declines in cognition and gait speed over time. Further studies on other cohorts are still needed to examine the reliability of the plasma biomarker cut-off values (identified in this study) for the classification of Aβ+NfL+ status. Based on the combined plasma Aβ+NfL+ status, the incidence of neurological disorders, such as dementia and AD, can be further studied. Moreover, the association between Aβ+NfL+ status and the structural changes of motor-related brain regions can be examined to understand if the physical function changes found in our study is related to changes in motor-related central nervous system.

## Data Availability

The datasets used and/or analysed during the current study are available from the corresponding author on reasonable request.

## Abbreviations

Aβ: Amyloid-β
CCS: Composite cognitive score
MAPT: Multidomain Alzheimer’s Preventive Trial
MMSE: Mini-Mental State Examination
NfL: Neurofilament light chain
PET: Positron emission tomography
